# Longitudinal changes in sensory impairments and subsequent falls and fall-related injuries among middle-aged and older adults: a pooled analysis of four prospective cohorts

**DOI:** 10.1186/s12889-025-25679-5

**Published:** 2025-12-05

**Authors:** Jing Nie, Bing Liao, Xiaoling Wang, Gerson Ferrari, Leandro F. M. Rezende, Yuan Qiu, Huan Tao, Rongrong Le

**Affiliations:** 1 Population Research Institute, Nanjing University of Posts and Telecommunications, Jiangsu 210023 Nanjing, China; 2 High-Quality Development Evaluation Research Institute, Nanjing University of Posts and Telecommunications, Jiangsu 210023 Nanjing, China; 3 Jiangsu High-Quality Development Comprehensive Evaluation Research Base, Nanjing University of Posts and Telecommunications, Jiangsu 210023 Nanjing, China; 4 Nursing department, Central People’s Hospital of Zhanjiang, Guangdong 524037 Zhanjiang, China; 5 Department of Wound Repair, the First Affiliated Hospital of Wenzhou Medical University, Zhejiang 325000 Wenzhou, China; 6 Department of Endocrinology, the First Affiliated Hospital of Wenzhou Medical University, Zhejiang 325000 Wenzhou, China; 7 Escuela de Ciencias de la Actividad Física, el Deporte y la Salud, Universidad de Santiago de Chile (USACH), Santiago, Chile; 8 Facultad de Ciencias de la Salud, Universidad Autónoma de Chile, Santiago, Chile; 9 Chronic Disease Epidemiology Research Center, Department of Preventive Medicine, Federal University of Sao Paulo, Sao Paulo, Brazil; 10 Faculty of Health Sciences, Universidad Autónoma de Chile, Providencia, Chile; 11 Department of Hematology, West China Hospital of Sichuan University, Sichuan 610041 Chengdu, China; 12 National Clinical Research Center for Ocular Diseases, Eye Hospital, Wenzhou Medical University, No. 270 West College Road, Zhejiang 325027 Wenzhou, China; 13 School of Optometry and Ophthalmology, Eye Hospital, Wenzhou Medical University, No. 270 West College Road, Zhejiang 325027 Wenzhou, China

**Keywords:** Sensory impairments, Falls, Longitudinal changes, Association

## Abstract

**Background:**

Visual and hearing impairments are recognized as modifiable risk factors for falls. However, evidence on the longitudinal effects of changes in sensory status on the risk of falls and fall-related injuries remains limited. This study aimed to examine the association between changes in sensory impairment status and the incidence of falls and fall-related injuries among middle-aged and older adults.

**Methods:**

Self-reported visual and hearing impairments were assessed at baseline and at a follow-up survey conducted two to three years later. The primary outcomes were incident falls and fall-related injuries. Modified Poisson regression models with robust standard errors were used to estimate risk ratios (RRs) and 95% confidence intervals (CIs).

**Results:**

A total of 22,287 participants were included in the main analysis. Compared with maintaining good sensory function, new-onset sensory impairments, recovery from sensory impairments, and persistent sensory impairments were associated with higher risks of falls and fall-related injuries. New-onset sensory impairments were associated with a 20% higher risk of falls (RR = 1.20, 95% CI: 1.13–1.26) and a 37% higher risk of fall-related injuries (RR = 1.37, 95% CI: 1.22–1.54). Recovery from sensory impairments was associated with a modestly increased risk of falls (RR = 1.08, 95% CI: 1.02–1.15) and fall-related injuries (RR = 1.21, 95% CI: 1.07–1.38), while persistent sensory impairments were associated with the highest risks (falls: RR = 1.24, 95% CI: 1.18–1.29; fall-related injuries: RR = 1.45, 95% CI: 1.32–1.60). When compared with persistent sensory impairments, recovery was associated with a significantly lower risk of falls (RR = 0.88, 95% CI: 0.83–0.93) and fall-related injuries (RR = 0.83, 95% CI: 0.74–0.94).

**Conclusions:**

Longitudinal changes in sensory impairments are strongly associated with the risk of falls and fall-related injuries. Recovery from sensory impairment appears to be linked to a reduced risk, underscoring the potential benefits of interventions aimed at restoring sensory function.

**Supplementary Information:**

The online version contains supplementary material available at 10.1186/s12889-025-25679-5.

## Introduction

Falls are among the leading causes of unintentional injury-related deaths, substantial health care expenditures, and disabilities, accounting globally for 16.7 million years of life lost (YLLs) and 19.3 million years lived with disability (YLDs), particularly among older adults [1–3]. Despite a decrease in age-standardized rates of incidence, prevalence, and disability-adjusted life years (DALYs), the Global Burden of Diseases, Injuries, and Risk Factors Study (GBD) 2017 indicates that the total number of deaths due to falls has nearly doubled over the past three decades [3]. Falls - a preventable cause of morbidity and mortality - can be mitigated through interventions targeting modifiable risk factors, including frailty, visual impairment, hearing loss, and pain [2].

Most studies have demonstrated that single visual impairment, single hearing impairment, and dual sensory impairment are associated with an elevated risk of falls in both cross-sectional and longitudinal analyses [4–6]. However, these associations often appear weaker in longitudinal studies compared to cross-sectional ones [7]. Despite this, limited data exist on whether changes in sensory impairment over time influence the risk of falls. In addition, some studies have focused solely on the association between sensory impairment with fall risk [8, 9], and few have examined whether changes in sensory impairment are associated with the risk of falls-related injuries. Addressing this gap could provide valuable insights for the primary prevention of falls and falls-related injuries in older adults with sensory impairments.

In this study, we analyzed data from four large prospective cohorts - the China Health and Retirement Longitudinal Study (CHARLS), the Health and Retirement Study (HRS), the English Longitudinal Study of Ageing (ELSA), and the Mexican Health and Aging Study (MHAS) - to examine the associations of new-onset sensory impairments, recovery from sensory impairments, and persistent sensory impairments with the risk of falls and fall-related injuries, compared to stable good sensory status. Furthermore, we explored whether recovery from sensory impairment was associated with reduced risks of falls and fall-related injuries compared with persistent sensory impairments. Additionally, we conducted subgroup analyses to assess these associations across sex, and age groups.

## Methods

### Data and study sample

Data were drawn from four international longitudinal cohorts focusing on middle-aged and older adults: the China Health and Retirement Longitudinal Study (CHARLS) [10], the English Longitudinal Study of Ageing (ELSA) [11], the Health and Retirement Study (HRS) [12], and the Mexican Health and Aging Study (MHAS) [13]. These surveys aim to provide comparable data to address the issue of population aging and have been described in details elsewhere [14]. Each cohort provides information on vision and hearing quality, as well as the incidence of falls and fall-related injuries.

For this study, data were selected from three survey waves within each cohort to ensure comparability and reliability of longitudinal data across similar timeframes. Data from CHARLS were drawn from wave 2 (2013) to wave 4 (2018). HRS data were taken from wave 12 (2014/2015) to wave 14 (2018/2019). MHAS data were from wave 4 (2015) to wave 6 (2021). ELSA data were from wave 7 (2014/2015) to wave 9 (2018/2019). The first wave from each cohort served as the baseline.

Participants aged ≥ 55 years were included. Individuals with missing data on vision or hearing quality at either the baseline or second wave, as well as those missing data on falls, were excluded. Those with missing data on other variables (marital status, educational level, wealth status, and multimorbidity) were also excluded. The final analytical sample comprised 22,287 participants.

All studies were approved by their respective national ethics committees, and written informed consent was obtained from all participants.

### Assessment of sensory impairment

Sensory impairment status was assessed through self-reported hearing and vision quality. For hearing, participants were asked: “Is your hearing [using a hearing aid if applicable] excellent, very good, good, fair, or poor?“. For vision, participants in HRS, ELSA, and MHAS were asked: “Is your eyesight [using glasses or corrective lenses if applicable] excellent, very good, good, fair, or poor?” Participants rating their vision or hear as excellent, very good, or good were classified as having no impairment. In CHARLS, vision quality was assessed using two questions: “How would you rate your eyesight for seeing things at a distance?” and “How would you rate your eyesight for seeing things up close, such as reading a newspaper?“. Those reporting excellent, very good, or good, for both distance or near vision were considered to have no visual impairment.

We combined hearing and vision impairments into a single sensory impairment variable to enhance interpretability and statistical power, particularly when assessing changes in sensory function over time. Sensory impairment status was categorized as: (1) no sensory impairments, and (2) sensory impairment (hearing only, vision only, or dual impairments). This classification was similar to that used in previous study [15].

Changes in sensory impairments between the baseline and the second survey wave were classified as: (1) persistently normal (no sensory impairments at both time points), (2) progression (transitioned from no impairment to impairment) (3) improvement (transitioned from impairment to no impairment), and (4) persistently impaired (impairment at both time points).

### Falls and fall-related injuries

The primary outcomes were the new onset of falls and fall-related injuries. Self-reported falls were identified by the question: “Have you fallen down in the last two years/since last interview?” (yes or no). Fall-related injuries were defined as injuries from a fall that required medical treatment.

### Covariates

Covariates included demographic characteristics, comorbidities, and lifestyle factors. To ensure valid cross-national comparisons, we used harmonized variables from the Gateway to Global Aging Data (g2aging.org), which applies rigorous cross-cohort harmonization protocols [14]. Demographic harmonized variables included age, sex, marital status, education, and wealth. Comorbidities were defined as self-reported physician diagnoses of hypertension, diabetes, cancer, heart disease, or stroke. Lifestyle factors included smoking status, drinking status, and body mass index (BMI). To ensure consistency across the four cohorts (CHARLS, ELSA, HRS, and MHAS), marital status was categorized into two groups: married or partnered and other (including separated, divorced, unmarried, or widowed). Following the International Standard Classification of Education (ISCED)–based harmonization standards, education was classified into three levels using standardized variables: less than high school, high school or some college, and college or higher. Total wealth was harmonized by aggregating comparable assets and liabilities into a unified measure of net worth, which was then categorized into low, middle, and high levels based on tertile cut-points of the overall wealth distribution. Smoking status was grouped into three categories: never smoked, former smoker, and current smoker. Drinking status was classified into two categories: never drinker or ever drinker. BMI was categorized according to the World Health Organization (WHO) guidelines: underweight (< 18.5 kg/m²), normal weight (18.5–24.9 kg/m²), overweight (25–29.9 kg/m²), and obesity (≥ 30 kg/m²). Comorbidities were grouped into three levels: no comorbidities, one comorbidity, and two or more comorbidities.

### Statistical analyses

Descriptive analyses were performed according to changes in sensory impairment status. We performed modified Poisson regression models with robust standard errors to estimate risk ratios (RRs) and 95% confidence intervals (CIs) for the association between changes sensory impairment status and the risk of falls (reference group: stable good sensory status) and fall-related injuries [16, 17] We fitted three models: Model 1adjusted for age and sex; Model 2 further adjusted for education, marital status, and wealth; Model 3 additionally adjusted for country and comorbidities.

To explore whether recovery of sensory impairment status was associated with lower risk of falls and fall-related injuries, we repeated analyses using persistent impairment as the reference category. Subgroup analyses were conducted by age (55–64 years and 65 years and older), sex (women and men), and country (China, United States, England and Mexico). Multiplicative interactions were tested using Wald test. We also examined changes in hearing-only and vision-only in relation to the risk of falls and fall-related injuries.

Sensitivity analyses were performed by: (a) adding lifestyle covariates to model 3, (b) applying national sample weights for each country and pooling results using a random-effects meta-analysis, (c) performing multiple imputation for missing covariates [18], and (d) Calculating E-values to assess the potential impact of unmeasured confounding [19]. All statistical analyses were conducted using STATA version 15.0, with statistical significance set at *P* < 0.05.

## Results

### Baseline characteristics of the study population

In the final analysis, a total of 22,287 participants were included. Based on sensory impairment status across two time points, 7621 participants (34.2%) consistently reported no sensory impairments, 2342 participants (10.5%) experienced remission of sensory impairment, 3047 participants (13.7%) experienced progression of sensory impairment, and 9277 participants (41.6%) had persistent sensory impairment (Table 1).

**Table 1:** Baseline characteristics of participants for changes in sensory impairment analyses

	Changes in sensory impairment status [*N* (%)]			
	Persistently normal	Improvement	Progression	Persistently impaired
Total	7621 (34.19)	2342 (10.51)	3047 (13.67)	9277 (41.63)
Age, years [mean (SD)]	70.01 (7.36)	68.79 (8.33)	69.30 (8.45)	68.23 (8.45)
Sex				
Male	2980 (30.89)	1051 (10.89)	1284 (13.31)	4333 (44.91)
Female	4641 (36.72)	1291 (10.21)	1763 (13.95)	4944 (39.12)
Marital status				
Married or partnered	5151 (33.24)	1572 (10.14)	2028 (13.09)	6745 (43.53)
Other marital status	2470 (36.37)	770 (11.34)	1019 (15.01)	2532 (37.28)
Education				
Below high school	2278 (17.91)	1449 (11.39)	1844 (14.49)	7151 (56.21)
High school or some college	3457 (53.93)	597 (9.31)	831 (12.96)	1525 (23.79)
College or above	1886 (59.78)	296 (9.38)	372 (11.79)	601 (19.05)
Wealth				
Low	1987 (26.44)	801 (10.66)	1032 (13.73)	3696 (49.18)
Middle	2653 (34.90)	813 (10.69)	1014 (13.34)	3122 (41.07)
High	2981 (41.58)	728 (10.15)	1001 (13.96)	2459 (34.30)
Multimorbidity				
No conditions	2465 (32.81)	754 (10.04)	951 (12.66)	3343 (44.50)
One condition	2861 (35.30)	846 (10.44)	1138 (14.04)	3260 (40.22)
At least two conditions	2295 (34.41)	742 (11.13)	958 (14.36)	2674 (40.10)
Falls (baseline)				
Yes	2091 (29.16)	849 (11.84)	1064 (14.84)	3167 (44.16)
No	5530 (36.58)	1493 (9.88)	1983 (13.12)	6110 (40.42)
Smoking status				
Never	3681 (32.42)	1180 (10.39)	1584 (13.95)	4909 (43.24)
Previous	3264 (42.72)	828 (10.84)	1057 (13.84)	2491 (32.60)
Current	605 (27.02)	268 (11.97)	311 (13.89)	1055 (47.12)
Missing	71 (6.74)	66 (6.26)	95 (9.01)	822 (77.99)
Drinking status				
Never	3008 (26.10)	1316 (11.42)	1703 (14.78)	5498 (47.70)
Ever	4448 (42.66)	995 (9.54)	1281 (12.29)	3703 (35.51)
Missing	165 (49.25)	31 (9.25)	63 (18.81)	76 (22.69)
BMI(kg/m^2^)				
<18.5	91 (22.30)	25 (6.13)	40 (9.80)	252 (61.76)
18.5–24.9	2054 (30.48)	626 (9.29)	874 (12.97)	3185 (47.26)
25-29.9	2895 (37.42)	889 (11.49)	1098 (14.19)	2855 (36.90)
≥30.0	2087 (40.70)	571 (11.13)	742 (14.47)	1728 (33.70)
Missing	494 (21.71)	231 (10.15)	293 (12.88)	1257 (55.25)

Participants with persistent sensory impaired were more likely to be men (44.9%), younger (mean age: 68.2 years), married or partnered (43.5%), and to have lower levels of education (56.2% had below high school education). They were also more likely to have lower wealth status (49.2%) and current smoker (47.1%). In addition, these participants were more likely to have underweight (61.8%) and to report no chronic conditions (44.5%).

### Association of changes in sensory impairment status with risk of falls and fall-related injuries

**Fig. 1:**
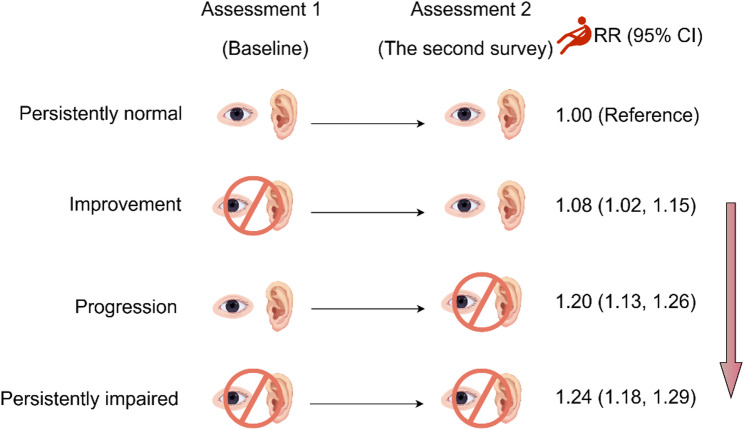
Associations of change in sensory impairment with falls (*n* = 22,287) (By Figdraw)

Figure 1 presents the associations between changes in sensory impairment status and the risk of falls. Compared with participants maintaining a stable good sensory status, those with persistent sensory impairments had a significantly higher risk of falls (RR = 1.24, 95% CI: 1.18–1.29, E-value = 1.79). Both recovery from sensory impairments and new-onset sensory impairments were associated with a higher risk of falls relative to stable good sensory status (recovery: RR = 1.08, 95% CI: 1.02–1.15, E-value = 1.37; new-onset: RR = 1.20, 95% CI: 1.13–1.26, E-value = 1.69).

**Fig. 2:**
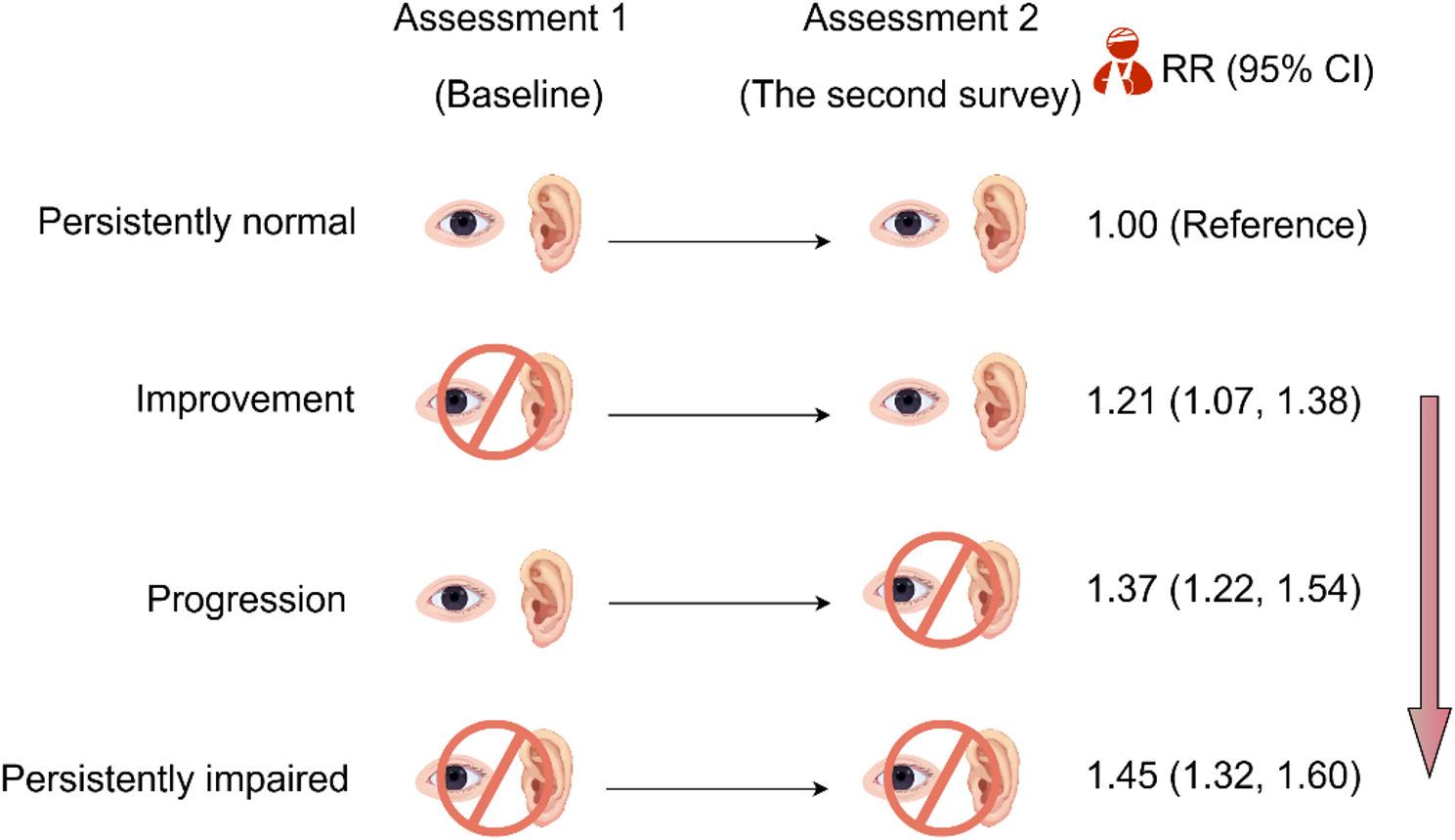
Associations of change in sensory impairment with fall-related injuries (*n* = 22,281) (By Figdraw). * Adjustments were made for baseline age, gender, education, marital status, wealth, falls at baseline, country and comorbidities

Persistent sensory impairments, recovery from sensory impairments, and new-onset sensory impairments were each significantly associated with increased risk of fall-related injuries (persistent: RR = 1.45, 95% CI: 1.32–1.60, E-value = 2.26; recovery: RR = 1.21, 95% CI: 1.07–1.38, E-value = 1.71; new-onset: RR = 1.37, 95% CI: 1.22–1.54, E-value = 2.08; Fig. 2). Compared with persistent sensory impairment, recovery was associated with lower risks of falls (RR = 0.88, 95% CI: 0.83–0.93) and fall-related injuries (RR = 0.83, 95% CI: 0.74–0.94).

### Association between changes in visual impairments or hearing impairments and risks of incident falls and falls injuries

In the fully adjusted model, persistent and new-onset visual impairment were significantly associated with higher risk of falls compared with stable good visual status (persistent: RR = 1.19, 95% CI: 1.14–1.25; new-onset: RR = 1.19, 95% CI: 1.13–1.26; Table 2). We found a trend toward an association between recovery from visual impairment and a higher risk of falls (RR = 1.06, 95% CI: 1.00–1.12).

**Table 2:** Associations of change in visual and hearing impairment with falls and fall-related injuries

Sensory impairment change		Events/Participants (%)	Visual impairment		Events/Participants (%)	Hearing impairment	
			RR (95%CI)	*P* value		RR (95%CI)	*P* value
Falls (*n* = 22,287)							
Persistently normal		3516/10,541 (33.4%)	1.00 [Reference]		3981/11,639 (34.2%)	1.00 [Reference]	
Improvement		881/2337 (37.7%)	1.06 (1.00, 1.12)	0.06	826/2169 (38.1%)	1.08 (1.02, 1.14)	0.01
Progression		1245/2975 (41.8%)	1.19 (1.13, 1.26)	<0.001	1133/2964 (38.2%)	1.12 (1.06, 1.18)	<0.001
Persistently impaired		2383/6434 (37.0%)	1.19 (1.14, 1.25)	<0.001	2085/5515 (37.8%)	1.18 (1.13, 1.23)	<0.001
Fall-related injuries (*n* = 22,281)							
Persistently normal		1097/10,538 (10.4%)	1.00 [Reference]		1272/11,635 (10.9%)	1.00 [Reference]	
Improvement		312/2336 (13.4%)	1.16 (1.02, 1.31)	0.02	304/2168 (14.0%)	1.22 (1.09, 1.37)	<0.001
Progression		454/2975 (15.3%)	1.34 (1.20, 1.49)	<0.001	426/2964 (14.4%)	1.29 (1.16, 1.43)	<0.001
Persistently impaired		909/6432 (14.1%)	1.32 (1.20, 1.46)	<0.001	770/5514 (14.0%)	1.30 (1.19, 1.42)	<0.001

Persistent visual impairment (RR = 1.32, 95% CI: 1.20–1.46), new-onset visual impairment (RR = 1.34, 95% CI: 1.20–1.49), and remission of visual impairment (RR = 1.16, 95% CI: 1.02–1.31) were each associated with an increased risk of fall-related injuries (Table 2). Compared with persistent visual impairments, recovery was associated with lower risks of falls (RR = 0.89, 95% CI: 0.84–0.94) and fall-related injuries (RR = 0.88, 95% CI: 0.78–0.99).

Persistent, new-onset, and remission from hearing impairment were all associated with higher risks of both falls (persistent: RR = 1.18, 95% CI: 1.13–1.23; recovery: RR = 1.08, 95% CI: 1.02–1.14; new-onset: RR = 1.12, 95% CI: 1.06–1.18) and fall-related injuries ((persistent: RR = 1.30, 95% CI: 1.19–1.42; recovery: RR = 1.22, 95% CI: 1.09–1.37; new-onset: RR = 1.29, 95% CI: 1.16–1.43; Table 2). Compared with persistent hearing impairments, recovery was significantly associated with reduced risk of falls (RR = 0.91, 95% CI: 0.86–0.97), but not with fall-related injuries (RR = 0.94, 95% CI: 0.83–1.06).

### Sensitivity analysis

Subgroup analysis indicated no significant multiplicative interaction between changes in sensory impairment status and age or sex for the risks of falls and fall-related injuries (all P-values > 0.05; Table 3). Adjusting for lifestyle factors did not substantially change the main findings (Table 4). Applying national sample weights for each country and pooling results using a random-effects meta-analysis also produced similar results (Table 5). The multiple imputation analysis for main results were similar (Data not shown). E-values (1.7–2.3) indicate robust findings requiring strong confounding to nullify associations.

**Table 3:** Subgroup analyses

Sensory impairment change	Falls					Fall-related injuries				
	RR (95%CI)	*P* value	RR (95%CI)	*P* value	*P* value for interaction	RR (95%CI)	*P* value	RR (95%CI)	*P* value	*P* value for interaction
**Age**	**55–64**		**65+**		0.50	**55–64**		**65+**		0.78
Persistently normal	1.00 [Reference]		1.00 [Reference]			1.00 [Reference]		1.00 [Reference]		
Improvement	1.14 (1.00, 1.30)	0.06	1.08 (1.01, 1.16)	0.03		1.42 (1.07, 1.87)	0.01	1.20 (1.03, 1.39)	0.02	
Progression	1.28 (1.13, 1.44)	<0.001	1.19 (1.12, 1.27)	<0.001		1.43 (1.10, 1.85)	0.01	1.42 (1.25, 1.61)	<0.001	
Persistently impaired	1.14 (1.03, 1.26)	0.01	1.20 (1.14, 1.26)	<0.001		1.49 (1.20, 1.86)	<0.001	1.49 (1.34, 1.65)	<0.001	
**Sex**	**man**		**woman**		0.62	**man**		**woman**		0.15
Persistently normal	1.00 [Reference]		1.00 [Reference]			1.00 [Reference]		1.00 [Reference]		
Improvement	1.06 (0.95, 1.17)	0.30	1.12 (1.04, 1.21)	<0.001		1.06 (0.82, 1.36)	0.65	1.34 (1.15, 1.56)	<0.001	
Progression	1.18 (1.08, 1.30)	<0.001	1.24 (1.16, 1.32)	<0.001		1.32 (1.06, 1.64)	0.01	1.47 (1.29, 1.68)	<0.001	
Persistently impaired	1.16 (1.08, 1.25)	<0.001	1.19 (1.12, 1.25)	<0.001		1.51 (1.27, 1.78)	<0.001	1.47 (1.32, 1.64)	<0.001	

**Table 4:** Associations of change in sensory impairment with falls and fall-related injuries (adjustments for lifestyle covariates)

Sensory impairment change	Falls		Fall-related injuries	
	RR (95%CI)	*P* value	RR (95%CI)	*P* value
Persistently normal	1.00 [Reference]		1.00 [Reference]	
Improvement	1.06 (0.99, 1.13)	0.07	1.20 (1.04, 1.37)	0.01
Progression	1.17 (1.11, 1.24)	<0.001	1.36 (1.21, 1.54)	<0.001
Persistently impaired	1.21 (1.15, 1.27)	<0.001	1.43 (1.30, 1.59)	<0.001

**Table 5:** Meta-analysis of associations of change in sensory impairment with falls and fall-related injuries

Sensory impairment change	Falls		Fall-related injuries	
	RR (95%CI)	*P* value	RR (95%CI)	*P* value
Persistently normal	1.00 [Reference]		1.00 [Reference]	
Improvement	1.09 (1.02, 1.16)	0.01	1.20 (1.05, 1.37)	0.01
Progression	1.19 (1.09, 1.30)	<0.001	1.37 (1.17, 1.60)	<0.001
Persistently impaired	1.23 (1.18, 1.29)	<0.001	1.44 (1.29, 1.60)	<0.001

## Discussion

This multi-country, population-based prospective cohort study demonstrated that persistent sensory impairments, recovery from sensory impairments, and new-onset sensory impairments were each associated with a higher risk of falls and fall-related injuries compared to stable good sensory status. The results for changes in either visual or hearing impairments were broadly consistent. In addition, participants who recovered from sensory impairments to good status had lower risk of fall and fall-related injuries compared with those with persistent sensory impairments.

### Compared with other studies

Previous studies have shown both cross-sectional and longitudinal associations between vision impairment and a higher risk of falls [4, 20, 21]. Furthermore, self-reported vision impairment has been linked to fall risk even after adjustment for objective visual acuity [20]. Hearing impairment has also been associated with increased risk of falls [22–24]. Both vision and hearing impairments are considered potentially modifiable risk factors for falls [2].

Sensory loss increases with age, and both vision impairment and hearing impairment are common in older populations. Most studies consistently show that dual sensory impairment – concurrent loss of vision and hearing - is associated with a higher risk of falls compared with good sensory status [7, 25, 26]. Although the associations of vision impairment alone or hearing impairment alone with incidence of falls are not always statistically significant, they generally indicated an increased risk [7, 25]. Potential mechanisms underlying the association between sensory impairment and falls or fall-related injuries may include impaired postural control, diminished functional balance, reduced attentional resources, cognitive decline, and other mediating pathways that may act independently or synergistically to increase the risk [2, 7, 26–30].

In addition, cultural attitudes toward sensory loss may influence the observed associations. For instance, nearly 40% of older adults in England do not report hearing difficulties; Hispanic elders often underuse hearing aids when impairment is perceived as minor; and many older adults in China normalize sensory decline as part of aging, delaying evaluation and treatment. Such cultural differences could modulate observed effect sizes, and highlight the need for future research to explore how sociocultural factors shape the relationship between sensory impairments and fall risk [25, 31–33].

Compared with previous studies, our findings confirm that all types of sensory impairment are associated with increased risk of falls. However, evidence has been lacking on whether remission of sensory impairment is associated with higher risk of falls, as well as on the magnitude of association for different type of sensory impairment [7, 25, 26]. Our study found that recovery from sensory impairments, new-onset sensory impairments, and persistent sensory impairments were each significantly associated with an increased risk of falls compared with stable good sensory status. In a previous study using CHARLS dataset, the logistic mixed model was applied to evaluate the longitudinal association between the time-varying sensory impairment and risk of falls [25]. That study also found that participants with time-varying sensory impairment had a higher risk of falls compared to those without sensory impairment during 7-year follow-up, which is consistent with our results. However, the magnitude of riks associated with persistent sensory loss in that study was higher (ORs ranging from 2.60 to 3.28) than in our study. This discrepancy may be partly due to longer follow-up period and use of a mixed model in the previous study.

Of note, our study also found that remission of sensory impairment was associated with lower risk of falls compared to persistent sensory impairment. Improved balance and control may play a key role in this association. However, individuals with sensory impairment remission still had a significantly higher risk of fall compared to those with consistently good sensory function.

One possible explanation is that individuals who recover from sensory impairment may retain residual risk factors for falls—such as persistent fear of falling—which could partly account for the elevated risk relative to those with stable good sensory status. Our findings indirectly suggest that therapeutic or interventional strategies targeting sensory impairments—such as early cataract surgery, use of hearing aids, and prescription glasses or contact lenses—may be effective fall-prevention measures in individuals with sensory loss [2].

Fall-related injuries—a major barrier to achieving healthy aging—were also the focus of our study. In the United States, approximately 25% of fall-related injuries occur from falls, whereas in Australia, around 75% of injury-related hospitalizations are due to falls, contributing substantially to the economic burden [34–36]. Both self-reported hearing and vision impairments have been identified as significant risk factors for fall-related injuries, consistent with our findings [37, 38].

The association between changes in sensory status and fall-related injury risk mirrored that observed for falls risk; however, the association was notably stronger for fall-related injuries, particularly for persistent sensory impairment. These results suggest that sensory impairment may not only increase the likelihood of falls but also worsen their severity, leading to a greater likelihood of injury.

### Strengths and limitations

Firstly, it was based on a large, multinational sample and employed a longitudinal design. Second, few previous studies have examined the association between changes in sensory impairment status and the risk of incident falls and fall-related injuries; to our knowledge, this is the first study to investigate this relationship, which carries important public health implications. Third, we performed an extensive set of subgroup and sensitivity analyses to test the robustness of our main findings.

However, several limitations should be acknowledged. First, given the observational nature of our study, causal inferences regarding the relationship between changes in sensory impairment status and the risk of falls or fall-related injuries should be made with caution. Second, there were differences in the definition of vision impairment between CHARLS and the other three surveys. Third, information on vision and hearing impairments was based on self-reports rather than objective measurements, which may introduce measurement error and misclassification bias. Previous studies have suggested that self-assessment may slightly overestimate the prevalence of visual impairment [39]. Fourth, falls and fall-related injuries may be underreported due to the retrospective nature of self-report, potentially leading to an underestimation of the true magnitude of association [7]. Moreover, our analysis was limited to non-fatal falls due to dataset constraints. Fifth, although we adjusted for multiple potential confounders, the possibility of residual confounding cannot be ruled out. Lastly, sample weights were not applied in the main analysis, which may limit the generalizability of our findings to nationally representative populations.

## Conclusions

Persistent sensory impairment, recovery from sensory impairment, and new-onset sensory impairment were all associated with a higher risk of falls and fall-related injuries compared with stable good sensory status. Participants who recovered from sensory impairment had a lower risk of incident falls and fall-related injuries compared with those who remained persistently impaired. Further research on falls and fall-related injuries is warranted to confirm our findings and elucidate the mechanisms underlying these associations. Such insights would provide an evidence-based foundation for developing targeted prevention programs and tailored interventions aimed at mitigating the risk of falls and fall-related injuries in individuals with sensory impairment.

## Supplementary Information

Supplementary Material 1.

## Data Availability

The current study utilized publicly available anonymized datasets.The ELSA data are freely available at https://beta.ukdataservice.ac.uk/datacatalogue/series/series?id=200011; The HRS data are freely available at https://hrs.isr.umich.edu/; The CHARLSdata are freely available at https://charls.pku.edu.cn/en/; The MHAS data are freely available at https://mhasweb.org.
