# Supplementary material for: Longitudinal changes in sensory impairments and subsequent falls and fall-related injuries among middle-aged and older adults: a pooled analysis of four prospective cohorts
Source: BMC Public Health. 2025 Dec 5;26:130. doi: 10.1186/s12889-025-25679-5 (PMC12797468; doi:10.1186/s12889-025-25679-5)
Supplement: Supplementary file 1 — Supplementary Material 1. [file 12889_2025_25679_MOESM1_ESM.docx]

**Supplementary Appendix**

**Table S1.** **Associations of change in sensory impairment with falls and fall-related injuries in different countries (Weighted).**

| **Sensory impairment change** | **CHARLS** | | **HRS** | | **ELSA** | | **MAHS** | |
| --- | --- | --- | --- | --- | --- | --- | --- | --- |
|  | **RR (95%CI)** | **P value** | **RR (95%CI)** | **P value** | **RR (95%CI)** | **P value** | **RR (95%CI)** | **P value** |
| **Falls** |  |  |  |  |  |  |  |  |
| Persistently normal | 1.00 [Reference] | | 1.00 [Reference] | | 1.00 [Reference] | | 1.00 [Reference] | |
| Improvement | 0.93 (0.55, 1.57) | 0.78 | 1.10 (0.96, 1.25) | 0.16 | 1.09 (0.93, 1.28) | 0.30 | 1.13 (0.92, 1.39) | 0.26 |
| Progression | 1.25 (0.78, 2.01) | 0.36 | 1.14 (1.02, 1.27) | 0.02 | 1.22 (1.06, 1.41) | 0.01 | 1.11 (0.93, 1.34) | 0.24 |
| Persistently impaired | 1.40 (0.94, 2.10) | 0.10 | 1.20 (1.10, 1.30) | ＜0.001 | 1.27 (1.13, 1.44) | ＜0.001 | 1.16 (1.00, 1.35) | 0.05 |
| **Fall-related injuries** |  |  |  |  |  |  |  |  |
| Persistently normal | 1.00 [Reference] | | 1.00 [Reference] | | 1.00 [Reference] | | 1.00 [Reference] | |
| Improvement | 0.72 (0.31, 1.67) | 0.44 | 1.34 (1.01, 1.78) | 0.04 | 1.51 (1.09, 2.09) | 0.01 | 1.57 (1.00, 2.44) | 0.05 |
| Progression | 1.22 (0.59, 2.50) | 0.59 | 1.64 (1.33, 2.02) | ＜0.001 | 1.39 (1.02, 1.91) | 0.04 | 1.04 (0.73, 1.49) | 0.82 |
| Persistently impaired | 1.23 (0.66, 2.27) | 0.52 | 1.37 (1.15, 1.64) | ＜0.001 | 1.76 (1.37, 2.26) | ＜0.001 | 1.37 (1.03, 1.81) | 0.03 |

*Adjustments were made for baseline age, gender, education, marital status, wealth, comorbidities and falls at baseline.

**Table S2. Associations of change in sensory impairment with falls and fall-related injuries in different countries**

| **Falls** | **CHARLS** | | **HRS** | | **ELSA** | | **MAHS** | |
| --- | --- | --- | --- | --- | --- | --- | --- | --- |
| **Sensory impairment change** | **RR (95%CI)** | **P value** | **RR (95%CI)** | **P value** | **RR (95%CI)** | **P value** | **RR (95%CI)** | **P value** |
| Persistently normal | 1.00 [Reference] | | 1.00 [Reference] | | 1.00 [Reference] | | 1.00 [Reference] | |
| Improvement | 0.96 (0.61, 1.51) | 0.87 | 1.02 (0.92, 1.14) | 0.69 | 1.10 (0.94, 1.28) | 0.22 | 1.15 (1.05, 1.26) | 0.003 |
| Progression | 1.31 (0.87, 1.96) | 0.20 | 1.12 (1.02, 1.23) | 0.02 | 1.14 (0.99, 1.31) | 0.07 | 1.29 (1.18, 1.40) | ＜0.001 |
| Persistently impaired | 1.52 (1.07, 2.16) | 0.02 | 1.19 (1.11, 1.27) | ＜0.001 | 1.26 (1.12, 1.42) | ＜0.001 | 1.26 (1.17, 1.35) | ＜0.001 |
| **Visual impairment change** |  |  |  |  |  |  |  |  |
| Persistently normal | 1.00 [Reference] | | 1.00 [Reference] | | 1.00 [Reference] | | 1.00 [Reference] | |
| Improvement | 0.90 (0.64, 1.27) | 0.55 | 0.99 (0.89, 1.11) | 0.88 | 1.14 (0.95, 1.36) | 0.17 | 1.11 (1.03, 1.20) | 0.01 |
| Progression | 1.25 (0.93, 1.68) | 0.14 | 1.18 (1.08, 1.30) | ＜0.001 | 1.23 (1.06, 1.43) | 0.01 | 1.21 (1.12, 1.30) | ＜0.001 |
| Persistently impaired | 1.37 (1.06, 1.76) | 0.02 | 1.09 (1.00, 1.18) | 0.04 | 1.37 (1.16, 1.61) | ＜0.001 | 1.20 (1.12, 1.28) | ＜0.001 |
| **Hearing impairment change** |  |  |  |  |  |  |  |  |
| Persistently normal | 1.00 [Reference] | | 1.00 [Reference] | | 1.00 [Reference] | | 1.00 [Reference] | |
| Improvement | 1.15 (0.92, 1.43) | 0.21 | 1.09 (0.98, 1.21) | 0.13 | 1.13 (0.97, 1.33) | 0.12 | 1.04 (0.96, 1.12) | 0.36 |
| Progression | 1.23 (1.01, 1.50) | 0.04 | 1.07 (0.97, 1.18) | 0.19 | 1.11 (0.95, 1.29) | 0.19 | 1.12 (1.05, 1.20) | ＜0.001 |
| Persistently impaired | 1.40 (1.19, 1.65) | ＜0.001 | 1.21 (1.12, 1.30) | ＜0.001 | 1.20 (1.05, 1.36) | 0.01 | 1.08 (1.02, 1.15) | 0.01 |
| **Fall-related injuries** |  |  |  |  |  |  |  |  |
| **Sensory impairment change** |  |  |  |  |  |  |  |  |
| Persistently normal | 1.00 [Reference] | | 1.00 [Reference] | | 1.00 [Reference] | | 1.00 [Reference] | |
| Improvement | 0.72 (0.34, 1.54) | 0.40 | 1.16 (0.92, 1.47) | 0.20 | 1.41 (1.04, 1.92) | 0.03 | 1.18 (0.97, 1.44) | 0.1 |
| Progression | 1.35 (0.71, 2.56) | 0.36 | 1.58 (1.32, 1.89) | ＜0.001 | 1.36 (1.01, 1.82) | 0.04 | 1.19 (0.99, 1.43) | 0.06 |
| Persistently impaired | 1.42 (0.81, 2.48) | 0.22 | 1.38 (1.18, 1.61) | ＜0.001 | 1.75 (1.38, 2.22) | ＜0.001 | 1.36 (1.16, 1.58) | ＜0.001 |
| **Visual impairment change** |  |  |  |  |  |  |  |  |
| Persistently normal | 1.00 [Reference] | | 1.00 [Reference] | | 1.00 [Reference] | | 1.00 [Reference] | |
| Improvement | 0.87 (0.50, 1.51) | 0.62 | 1.18 (0.94, 1.48) | 0.16 | 1.65 (1.19, 2.29) | ＜0.001 | 1.08 (0.91, 1.28) | 0.35 |
| Progression | 1.43 (0.90, 2.30) | 0.13 | 1.52 (1.26, 1.83) | ＜0.001 | 1.49 (1.09, 2.03) | 0.01 | 1.17 (1.00, 1.36) | 0.05 |
| Persistently impaired | 1.26 (0.83, 1.91) | 0.27 | 1.28 (1.07, 1.53) | 0.01 | 1.61 (1.15, 2.27) | 0.01 | 1.28 (1.11, 1.46) | ＜0.001 |
| **Hearing impairment change** |  |  |  |  |  |  |  |  |
| Persistently normal | 1.00 [Reference] | | 1.00 [Reference] | | 1.00 [Reference] | | 1.00 [Reference] | |
| Improvement | 1.00 (0.68, 1.47) | 1.00 | 1.13 (0.88, 1.44) | 0.34 | 1.26 (0.90, 1.75) | 0.17 | 1.32 (1.12, 1.54) | ＜0.001 |
| Progression | 1.55 (1.13, 2.12) | 0.01 | 1.27 (1.04, 1.55) | 0.02 | 1.17 (0.85, 1.62) | 0.33 | 1.22 (1.05, 1.42) | 0.01 |
| Persistently impaired | 1.39 (1.06, 1.83) | 0.02 | 1.30 (1.10, 1.53) | ＜0.001 | 1.56 (1.22, 2.00) | ＜0.001 | 1.22 (1.07, 1.40) | ＜0.001 |

*Adjustments were made for baseline age, gender, education, marital status, wealth, comorbidities and falls at baseline.

**Table S3. Associations of change in sensory impairment with falls and fall-related injuries compared with persistent sensory impairments.**

| **Sensory impairment change** | **Falls** | | **Fall-related injuries** | | **Visual impairment** | | | | **Hearing impairment** | | | |
| --- | --- | --- | --- | --- | --- | --- | --- | --- | --- | --- | --- | --- |
|  |  |  |  |  | **Falls** | | **Fall-related injuries** | | **Falls** | | **Fall-related injuries** | |
| Persistently normal | 0.81 (0.77, 0.85) | ＜0.001 | 0.69 (0.63, 0.76) | ＜0.001 | 0.84 (0.80, 0.88) | ＜0.001 | 0.76 (0.69, 0.83) | ＜0.001 | 0.85 (0.81, 0.89) | ＜0.001 | 0.77 (0.70, 0.84) | ＜0.001 |
| Improvement | 0.88 (0.83, 0.93) | ＜0.001 | 0.83 (0.74, 0.94) | ＜0.001 | 0.89 (0.84, 0.94) | ＜0.001 | 0.88 (0.78, 0.99) | 0.03 | 0.91 (0.86, 0.97) | ＜0.001 | 0.94 (0.83, 1.06) | 0.31 |
| Progression | 0.97 (0.92, 1.02) | 0.21 | 0.94 (0.85, 1.04) | 0.27 | 1.00 (0.95, 1.05) | 0.97 | 1.01 (0.91, 1.12) | 0.84 | 0.95 (0.90, 1.00) | 0.07 | 0.99 (0.89, 1.10) | 0.83 |
| Persistently impaired | 1.00 [Reference] | | 1.00 [Reference] | | 1.00 [Reference] | | 1.00 [Reference] | | 1.00 [Reference] | | 1.00 [Reference] | |

*Adjustments were made for baseline age, gender, education, marital status, wealth, falls at baseline, country and comorbidities.
